# A Case Report of Human Cutaneous Neosartorya hiratsukae Infection

**DOI:** 10.7759/cureus.69012

**Published:** 2024-09-09

**Authors:** Atithi Patel, Soham Patel, Alecia Blaszczak, Karen Krueger, Paras Vakharia

**Affiliations:** 1 Dermatology, Northwestern University Feinberg School of Medicine, Chicago, USA; 2 Infectious Disease, Northwestern University Feinberg School of Medicine, Chicago, USA

**Keywords:** aspergillus, fungal infection, israel, neosartorya hiratsukae, next generation sequencing (ngs), voriconazole

## Abstract

We present a case of a human cutaneous infection caused by *Neosartorya hiratsukae* in a 19-year-old male on adalimumab. While on a trip to Israel, the patient sustained a left knee abrasion after a fall while hiking, and subsequently went swimming in the Red Sea. The patient gradually developed a large, non-healing, erythematous, ulcerated plaque on the left knee.

Initial biopsy and tissue cultures were negative for infection, but due to a high suspicion for infection, further diagnostic testing was conducted. Broad range polymerase chain reaction and next-generation sequencing was performed, and 28S rDNA sequencing was positive for Neosartorya hiratsukae, a rare infectious agent in humans. This case highlights the importance of considering uncommon sources, such as fungal etiologies, in the differential diagnosis of non-resolving lesions, particularly in immunosuppressed patients. Furthermore, this case emphasizes the significance of advanced molecular techniques in identifying uncommon pathogens.

## Introduction

*Neosartorya hiratsukae*, a telemorph of *Aspergillus hiratsukae*, is a rare fungal species that has only been reported to be associated with infections in a few cases [1-6]. Non-cutaneous human infections reported include cerebral aspergillosis [2], rhinosinusitis [3], and peritonitis [4,5]. Infections with this organism are rare in immunocompetent patients. It is believed to be an opportunistic pathogen, occurring in immunocompromised patients [1]. This pathogen is often not identified or misidentified as other *Aspergillus* subtypes [1]. However, proper identification is vital, as treatments vary due to differing susceptibilities. Identification via routine laboratory testing is often insufficient, and requires molecular testing [1]. To date, no human cutaneous infections with *Neosartorya hiratsukae* have been reported. We report a case of human cutaneous infection caused by *Neosartorya hiratsukae* in a young 19-year-old immunosuppressed male.

## Case presentation

**Figure 1:**
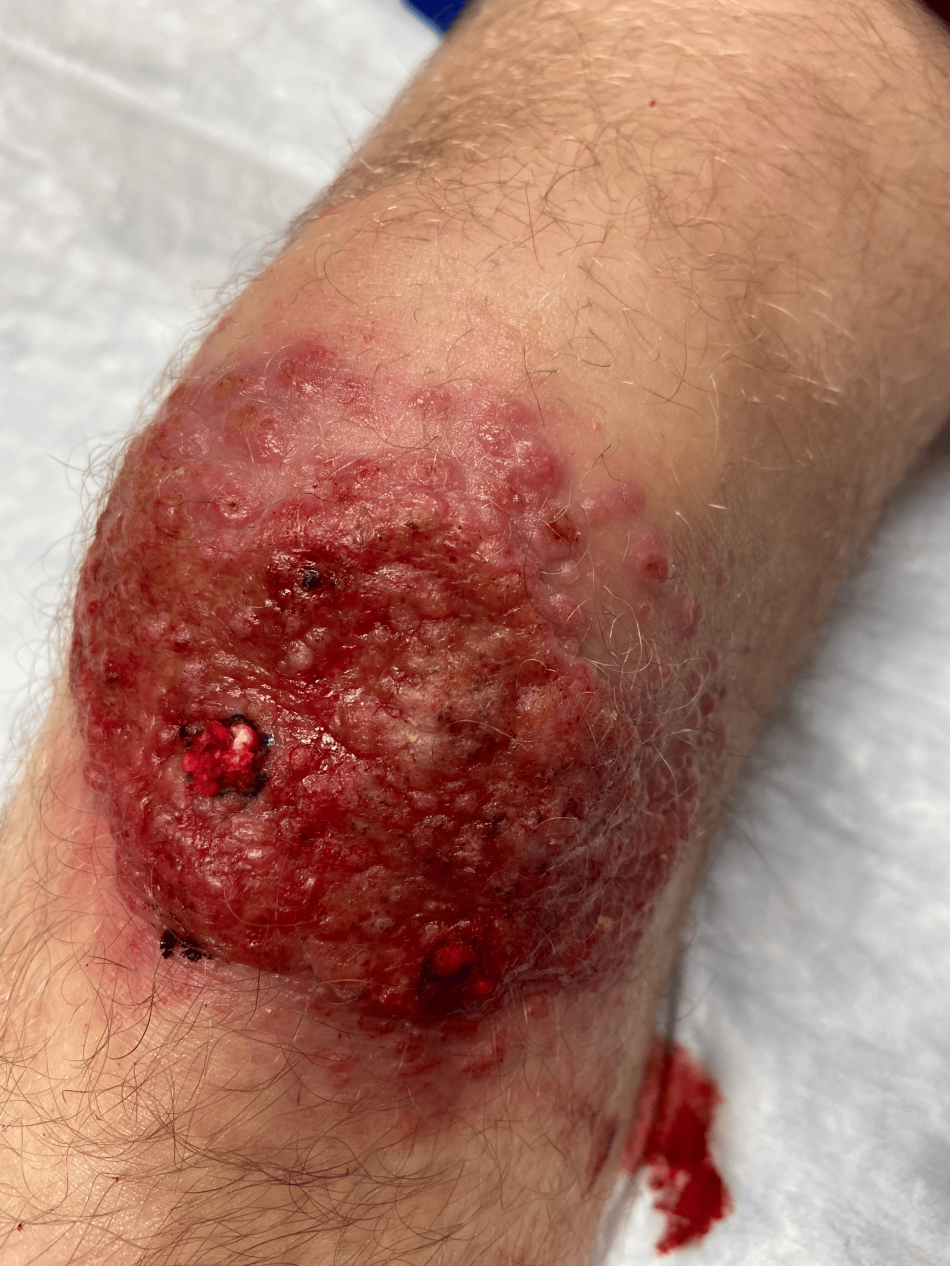
Left knee rash

A 19-year-old male with an outside history of cystic acne on adalimumab presented to our dermatology clinic with a progressive rash on his left knee. Approximately four months prior to presentation, the patient scraped his knee while hiking in Israel. After he scraped his knee, he also went swimming in the Red Sea. He subsequently developed a large, non-healing, erythematous, ulcerated plaque on the left knee, as seen in Figure 1 (photo taken after biopsy).

He denied any associated joint pain, was able to ambulate without issue, and denied any other systemic symptoms. Prior to presentation, the patient was treated with many topical and oral medications including triamcinolone 0.1% ointment, gentamicin 0.1% cream, mupirocin 2% cream, oral doxycycline and oral trimethoprim-sulfamethoxazole. The patient also saw orthopedic surgery after undergoing an MRI of the left knee which demonstrated a possible underlying joint effusion

**Figure 2:**
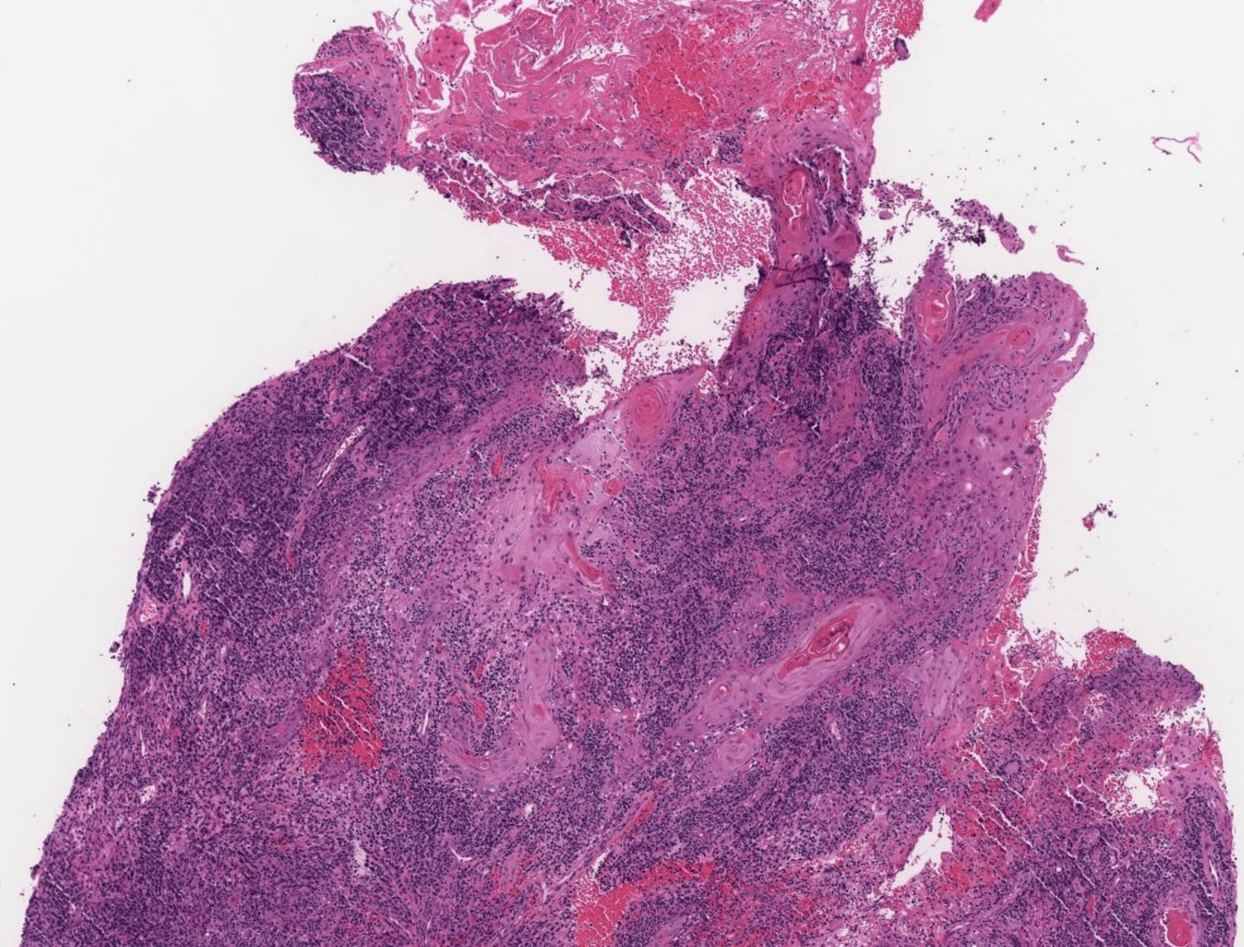
Histopathology Sections reveal a markedly corrugated epidermis with irregular proliferative epithelial hyperplastic and reactive changes without evidence of nuclear atypia. Some spongiosis and neutrophilic/lymphocytic exocytosis is noted. The process extends into the mid reticular dermis. The adjacent dermis shows mixed inflammation including granulomatous inflammation with multinucleated giant cells and plasma cells.

Given the clinical presentation and concern for infection, adalimumab treatment was stopped and punch biopsies were performed. Histopathological examination showed granulomatous dermatitis with pseudoepitheliomatous hyperplasia, as seen in Figure 2.

Tissue cultures obtained from the biopsy specimen were negative for bacterial, mycobacterial, and fungal organisms. However, due to the clinical suspicion of an infectious etiology, additional testing was pursued. Subsequent send-out testing from formalin-fixed paraffin-embedded for polymerase chain reaction (PCR) and next-generation sequencing was positive on 28S rRNA sequencing for *Neosartorya hiratsukae*. The patient was started on voriconazole 200mg twice daily which was increased to 300mg twice daily based on blood voriconazole levels (goal: 0.5 - 5.0 µg/mL), and treatment was continued until resolution of rash approximately five months later.

## Discussion

*Neosartorya hiratsukae *is a species in the genus *Aspergillus* and in the section Fumigati [1]. This species was first identified in 1991 and has been isolated from air and aloe juice. The first published case of human infection was in 2002 [2]. It was isolated from a patient in Brazil with cerebral aspergillosis. The patient was treated with itraconzole based on susceptibility testing with initial improvement but ultimately died from multiorgan failure. Additional cases of human infection have been reported including fungal rhinosinusitis and fungal peritonitis [3-5]. Minimum inhibitory concentration testing has been completed on the aforementioned isolates which have demonstrated variable resistance patterns. In all cases, isolates have been sensitive to voriconazole. In most cases, the patients were immunocompromised or immunosuppressed suggesting that this species is more likely to cause an opportunistic infection. The only reported cutaneous infection to date is in a hedgehog that presented with an alopecic patch with underlying scale and pinpoint hemorrhages [6]. In all case reports, they commented on the likely underreporting of this infectious agent as they are slow-growing white colonies that are often discarded as likely contaminants.

Our case is among the first reported cases, if not the first, of cutaneous infection in a human. Our patient was immunosuppressed in the setting of adalimumab therapy for reported treatment of cystic acne. It is possible the inoculation came from trauma and cutaneous exposures suffered during his travel. Holding his immunosuppression and treatment with oral voriconazole allowed for clinical resolution. Voriconazole was selected based on prior case reports [3-5], as well as in-vitro studies [7]. Treatment was targeted to our institution's reference range of goal blood levels, and continued until resolution of the rash per infectious disease recommendations. Our case also highlights the importance of additional diagnostic infectious disease testing in the setting of high clinical suspicion for infection. Advanced molecular techniques, such as PCR and next-generation sequencing, available at specialized reference laboratories, may be necessary to identify uncommon pathogens and ensure accurate diagnosis and appropriate management.

## Conclusions

This case emphasizes the importance of considering uncommon etiologies in the differential diagnosis of non-resolving cutaneous lesions, particularly in individuals with a history of outdoor activities in high-risk areas. Advanced molecular techniques may be crucial to pathogen identification in the correct clinical context. Dermatologists and healthcare professionals should maintain a high index of suspicion for fungal infections even if the initial workup is negative, and pursue further diagnostic testing when clinical suspicion persists. Increased awareness and understanding of this rare infection can contribute to timely diagnosis and appropriate management.
